# Analyses of MicroRNA and mRNA Expression Profiles Reveal the Crucial Interaction Networks and Pathways for Regulation of Chicken Breast Muscle Development

**DOI:** 10.3389/fgene.2019.00197

**Published:** 2019-03-18

**Authors:** Yuanfang Li, Yi Chen, Wenjiao Jin, Shouyi Fu, Donghua Li, Yanhua Zhang, Guirong Sun, Ruirui Jiang, Ruili Han, Zhuanjian Li, Xiangtao Kang, Guoxi Li

**Affiliations:** ^1^College of Animal Science and Veterinary Medicine, Henan Agricultural University, Zhengzhou, China; ^2^Henan Innovative Engineering Research Center of Poultry Germplasm Resource, Henan Agricultural University, Zhengzhou, China

**Keywords:** chicken, microRNA, mRNA, RNA-seq, breast muscle

## Abstract

There is a lack of understanding surrounding the molecular mechanisms involved in the development of chicken skeletal muscle in the late postnatal stage, especially in the regulation of breast muscle development related genes, pathways, miRNAs and other factors. In this study, 12 cDNA libraries and 4 small RNA libraries were constructed from Gushi chicken breast muscle samples from 6, 14, 22, and 30 weeks. A total of 15,508 known transcripts, 25,718 novel transcripts, 388 known miRNAs and 31 novel miRNAs were identified by RNA-seq in breast muscle at the four developmental stages. Through correlation analysis of miRNA and mRNA expression profiles, it was found that 417, 370, 240, 1,418, 496, and 363 negatively correlated miRNA–mRNA pairs of *W14* vs. *W6*, *W22* vs. *W6*, *W22* vs. *W14*, *W30* vs. *W6*, *W30* vs. *W14*, and *W30* vs. *W22* comparisons, respectively. Based on the annotation analysis of these miRNA–mRNA pairs, we constructed the miRNA–mRNA interaction network related to biological processes, such as muscle cell differentiation, striated muscle tissue development and skeletal muscle cell differentiation. The interaction networks for signaling pathways related to five KEGG pathways (the focal adhesion, ECM-receptor interaction, FoxO signaling, cell cycle, and p53 signaling pathways) and PPI networks were also constructed. We found that *ANKRD1*, *EYA2*, *JSC*, *AGT*, *MYBPC3*, *MYH11*, *ACTC1*, *FHL2*, *RCAN1*, *FOS*, *EGR1*, and *FOXO3*, *PTEN*, *AKT1*, *GADD45*, *PLK1*, *CCNB2*, *CCNB3* and other genes were the key core nodes of these networks, most of which are targets of miRNAs. The FoxO signaling pathway was in the center of the five pathway-related networks. In the PPI network, there was a clear interaction among *PLK1* and *CDK1*, *CCNB2*, *CDK1*, and *GADD45B*, and *CDC45*, *ORC1* and *MCM3* genes. These results increase the understanding for the molecular mechanisms of chicken breast muscle development, and also provide a basis for studying the interactions between genes and miRNAs, as well as the functions of the pathways involved in postnatal developmental regulation of chicken breast muscle.

## Introduction

In chickens, breast muscle is a major contributor to skeletal muscle and is directly correlated with meat quantity and quality. Therefore, determining the molecular mechanisms underlying skeletal muscle development has been a focus of research in the field of poultry genetic breeding.

Skeletal muscle development is a complex and precise process regulated by many genes, transcription factors, ncRNAs and signaling pathways. These factors form many interaction networks *in vivo* that regulate chicken skeletal muscle development (Mancinelli et al., 2012). Firstly, many interactions exist between genes involved in myoblast proliferation and differentiation (Huang et al., 2018). For example, insulin-like growth factor 1 (IGF-I) plays critical roles in skeletal myogenesis (Hamarneh et al., 2015), and the addition of *IGF-1* significantly upregulates genes encoding myogenic factors, such as *c-Myc* (Yu et al., 2015). Moreover, after decreased F-box protein 32 (FBXO32) expression, growth-related genes including pyruvate dehydrogenase kinase 4 (PDK4), insulin-like growth factor 2 receptor (IGF2R) and insulin growth factor-2 binding protein 3 (IGF2BP3) are significantly down-regulated (Chen et al., 2015). Secondly, many interactions exist between miRNAs and mRNAs during skeletal muscle development. miRNAs are endogenous non-coding RNAs that regulate gene expression at the post-transcriptional level by binding to the 3′-UTRs of target mRNAs. Many myogenic transcription factors and genes are regulated by various miRNAs. For instance, muscle differentiation-related miRNAs (MyomiRs) such as miR-1, miR-206, and miR-133, interact with myogenic genes, such as myogenin (MyoG), myogenic differentiation (MyoD), myogenic factor 5 (Myf5), myocyte enhancer factor 2 (MEF2) and paired box 7 (PAX7). These interactions play an important regulatory role in the biological processes involved in muscle development, such as muscle fiber type determination, muscle cell proliferation and differentiation, and skeletal muscle hypertrophy and atrophy (Nakasa et al., 2010; Moncaut et al., 2013; Badodi et al., 2015). Furthermore, many MyomiRs regulate the expression of *c-Myc*, such as miR-130a, miR-124, and miR-199 (Alexander et al., 2013; Ma et al., 2014; Qadir et al., 2014). Moreover, miR-222a and miR-126-5p inhibit the expression of their target genes cytoplasmic polyadenylation element-binding protein 3 (CPEB3) and fibroblast growth factor receptor 3 (FGFR3) in DF-1 cells, and they play roles in the regulation of embryonic muscle development and growth (Jebessa et al., 2018).

However, controlling chicken skeletal muscle growth is a highly complicated process involving multi-interaction relationships between miRNAs and mRNAs within an intricate signaling pathway, rather than a single interaction network. For instance, *MYOD1* expression is regulated by miR-1 via the mTOR signaling pathway (Sun et al., 2010). Moreover, miR-133-mediated Gli3 silencing, regulated by the Hedgehog pathway, orchestrates embryo myogenesis (Mok et al., 2018). In addition, myocyte-specific enhancer-binding factor 2 (MEF2C) interacts with the Notch3 and p38 MAPK pathways in regulating skeletal muscle differentiation (Zetser et al., 1999; Gagan et al., 2012). Inhibition of the expression of miR-21 down-regulates B-cell lymphoma-2 (Bcl-2), cyclinD1 and phosphorylated-protein kinase B (AKT), and up-regulates phosphatase and tensin homolog (PTEN) and bcl-associated protein X (Bax) (Li et al., 2018). These studies suggest that skeletal muscle development is regulated by complex interaction networks of genes, miRNAs and signaling pathways. Therefore, it is important to determine the interaction network of genes, miRNAs and signaling pathways in the regulation of skeletal muscle development. The current understanding of this network is lacking.

Chicken is an important agricultural animal in which, as in other animals, genetic factors are the main players affecting muscle development. For example, the heritability of muscle fiber number in chicken pectoralis superficialis is 0.43–0.48, and the heritability of muscle fiber size is 0.30–0.50 (Fu et al., 2018). Therefore, to improve poultry genetics and production, it is important to clarify the interaction mechanisms among genes, miRNAs and pathways in chicken skeletal muscle, especially in the development of breast muscle. Several reports have described gene regulation, miRNA identification and pathway regulation in chicken skeletal muscle development. For instance, miR-203 has been found to inhibit the expression of *c-JUN* and *MEF2C*, thereby inhibiting muscle proliferation and differentiation in chicken myoblasts (Luo et al., 2014). Moreover, *c-JUN* inhibits p53 expression and induces cell cycle progression (Schreiber et al., 1999). However, these studies have been limited to the roles of genes or miRNAs or pathways themselves in chicken skeletal muscle development, and there is a lack of understanding of the interaction of these factors in chicken skeletal muscle development. In addition, early studies have focused on chicken embryos and early postnatal periods, but the understanding of the postnatal late development of chicken skeletal muscle remains lacking.

Gushi chicken is an excellent variety of egg- and meat-providing chickens native to Gushi County, Henan Province, China. This variety has many favorable characteristics: the meat is tender, tasty, and has a fresh unique flavor. Gushi chicken is suitable for breeding and production. Many studies have been conducted on the molecular mechanisms of resource conservation, strain selection, industrial production and trait formation of this local variety. Although the Gushi chicken has many excellent features, its growth rate is slow. The molecular mechanisms related to muscle development regulation remain unclear in the Gushi chicken. In order to reveal the crucial molecular mechanisms and molecular networks underlying muscle development in this variety, in the present study, we identified miRNA and mRNA expression profiles in chickens at four different stages (6, 14, 22, and 30 weeks) representing chicks, early young chickens, late young chickens, and laying hens, respectively. The key negatively correlated miRNA–mRNA interaction networks and pathways associated with breast muscle development were identified. Our aim was to aid in understanding the molecular mechanisms of late developmental regulation of chicken skeletal muscle, to provide a basis for further study of the interactions among miRNAs, mRNAs and signaling pathways in chicken skeletal muscle development. Our study also provides basic data for Gushi chicken trait mining and resource conservation.

## Materials and Methods

### Ethics Statement

All animal experiments were performed according to protocols and guidelines approved by the Institutional Animal Care and Use Committee of Henan Agricultural University, China.

### Sample Collection and RNA Isolation

Female native Chinese chickens, known as Gushi chickens, were used in this study. The raising method was the same as in our previous research (Fu et al., 2018). Briefly, the chickens were fed with a two-phase feeding system, in which the first phase (before 14 weeks) comprised 18.5% crude protein and 12.35 MJ/kg, and the second phase (after 14 weeks) comprised 15.6% crude protein and 12.75 MJ/kg, and the chickens were given free access to water. The left breast muscle tissues were collected from chickens at 6, 14, 22, and 30 weeks of age, then frozen immediately in liquid nitrogen and stored at -80°C until RNA extraction.

Total RNA for RNA sequencing (RNA-seq) was isolated from 12 breast muscle samples (three each from 6-, 14-, 22-, and 30-week samples) with TRIzol reagent (Invitrogen, Carlsbad, CA, United States) according to the manufacturer’s protocol. The purity and concentration of the RNA samples were evaluated on a NanoDrop 2000 spectrophotometer (Thermo Scientific, Wilmington, DE, United States), and standard denaturing agarose gel electrophoresis was used to monitor the degradation and contamination. The RNA integrity was assessed with an RNA Nano 6000 Assay Kit of the Agilent Bioanalyzer 2100 system (Agilent Technologies, Palo Alto, CA, United States).

### Illumina Deep Sequencing and Sequence Analysis of mRNA

A total of 12 cDNA libraries were constructed with the breast muscle tissues from Gushi chickens in the four developmental stages (6, 14, 22, and 30 weeks). A total of 3 μg total RNA per sample was used as input material for the cDNA library. First, ribosomal RNA was removed with an Epicentre Ribo-Zero^TM^ rRNA Removal Kit (Epicentre, Madison, WI, United States), and rRNA free residues were cleaned up through ethanol precipitation. Subsequently, sequencing libraries were generated using the rRNA-depleted RNA by NEBNext^®^ Ultra^TM^ Directional RNA Library Prep Kit for Illumina^®^ (NEB, Ipswich, MA, United States), by following manufacturer’s recommendations. Finally, products were purified (AMPure XP system), and library quality was assessed on an Agilent Bioanalyzer 2100 system. The libraries were sequenced on an Illumina Hiseq 2500 platform, and 150 bp paired-end reads were generated.

Raw data in fastq format were first processed through in-house scripts. The Illumina sequencing raw reads were obtained by removal of reads containing adapter, reads containing ploy-N and low-quality reads from raw data, in which the number of bases with a quality value *Q* ≤20 was >50%. The Q20, Q30, and GC content of the clean data were calculated. All down-stream analyses were based on the clean data, which were of high quality. Reference genome and gene model annotation files were downloaded directly from the genome website. The index of the reference genome was built using Bowtie v2.2.3, and paired-end clean reads were aligned to the reference genome using TopHat v2.0.12. The Cufflinks v2.1.1 Reference Annotation Based Transcript (RABT) assembly method was used to construct and identify both known and novel transcripts from the TopHat alignment results. The fragments per kilobase per million reads (FPKM) (Trapnell and Salzberg, 2009) for each gene was calculated on the basis of the length of the gene and read counts mapped to the gene.

### Illumina Deep Sequencing and Sequence Analysis of miRNA

Four small RNA libraries were constructed by using the breast muscle tissues from Gushi chickens at the developmental stages of 6 weeks (*W6*), 14 weeks (*W14*), 22 weeks (*W22*), and 30 weeks (*W30*), with a NEBNext^®^ Multiplex Small RNA Library Prep Set for Illumina^®^ (NEB). Equal amounts of high-quality RNA samples from three individuals in each developmental stage were mixed with 3 μg RNA sample, which was used as input material for construction of the small RNA library. The quality and quantity of the cDNA library were assessed using a Qubit2.0 (Life Technologies) and an Agilent Bioanalyzer 2100 system, respectively, and the effective concentrations in the libraries were greater than 2 nM. Finally, the cDNA library preparations were sequenced using the Illumina HiSeq2500 sequencing platform, and 50 bp single-end reads were generated.

The raw sequencing reads were processed by evaluating the sequencing quality, calculating the length distribution of small RNA reads and removing low quality reads and adaptor sequences. Clean high-quality reads with lengths of 18–35 nt were used in the subsequent analyses. The small RNA tags were mapped to reference sequences in Bowtie2^1^. Mapped small RNA tags were used to search for known miRNAs. miRBase22.0 was used as a reference, and the modified software mirdeep2 (Friedlander et al., 2012) and srna-tools-cli were used to obtain the potential miRNAs and draw the secondary structures. To remove tags originating from protein-coding genes, repeat sequences, rRNAs, tRNAs, snRNAs, and snoRNAs, small RNA tags were mapped to the RepeatMasker or Rfam databases or data from the specified species itself. The characteristics of the hairpin structures of miRNA precursors can be used to predict novel miRNA. The software miREvo (Friedlander et al., 2012; Wen et al., 2012) mirdeep2 were used to predict novel miRNA through exploring the secondary structures, Dicer cleavage sites and the minimum free energy of the small RNA tags unannotated in the former steps.

### Differential Expression Analysis

The expression levels of mRNAs in the different libraries constructed from breast muscle were estimated on the basis of the Illumina sequencing data, according to the FPKM values. The FC (fold change) for each mRNA between two discretionary stages was calculated according to comparisons of six combinations; that is, *W14* vs. *W6*, *W22* vs. *W14*, *W22* vs. *W6*, *W30* vs. *W6*, *W30* vs. *W14*, and *W30* vs. *W22*. Differential expression analysis of two groups was performed with the DESeq R package (1.8.3). Genes with adjusted *p* < 0.05 and |FC| > 1 found by DESeq were considered differentially expressed.

In addition, based on the values of normalized transcripts per kilobase per million reads (TPM), the DEGseq R package was used to analyze the differentially expressed miRNAs (DEMs). *q*-values were adjusted by *p*-values. A *q*-values < 0.01 and |FC| > 1 was set as the threshold for significantly DEMs by default.

### Gene Ontology and Kyoto Encyclopedia of Genes and Genomes Analyses

The mRNAs and enriched genes among miRNA targeted mRNAs were annotated and classified by Gene Ontology (GO^2^) with the software GOSeq (Release2.12) (Young et al., 2010) and Kyoto Encyclopedia of Genes and Genomes (KEGG^3^) pathway analysis with the software KOBAS (v2.0) (Mao et al., 2005) to visualize data. Only GO terms or KEGG pathways with corrected *p*-values (*t*-test) < 0.05 were considered to indicate significant enrichment.

### miRNA–mRNA Interaction Analysis

The miRNA and transcriptome profiles of the four developmental stages were constructed using the same breast muscle samples from Gushi chickens. To analyze the interactions between miRNAs and mRNAs, first, miRanda (Enright et al., 2003) was used to predict miRNA target genes two with psRobot_tar in psRobot (Wu et al., 2012). Then miRNA–mRNA interactions were calculated according to miRNA expression profiles and transcriptome data, and negatively correlated miRNA–mRNA pairs were determined using Pearson correlation analysis. Finally, mRNA and miRNA expression data were integrated using the MAGIA network tool, and the network was then constructed by Cytoscape.

### Quantitative Real-Time PCR (qRT-PCR) Analysis

For the qRT-PCR analysis of genes, reverse transcription was performed using a PrimerScript^TM^ RT reagent Kit (TaKaRa, Dalian, China) according to the manufacturer’s instructions. qRT-PCR with iTaq^TM^ Universal SYBR^®^ Green Supermix Kit (Bio-Rad Laboratories Inc., Waltham, MA, United States) was performed with a LightCycler^®^ 96 instrument qRT-PCR system (Roche, Basel, Switzerland) as follows: 95°C for 3 min; 40 cycles of 95°C for 10 s, annealing at 60°C for 30 s, and 72°C for 30 s, and 72°C for 1 min. The data were analyzed with the 2^-ΔΔCt^ method. The chicken *GAPDH* gene was used as a reference gene for normalization of target gene data. The sequences of qRT-PCR primers are listed in Supplementary Table S1.

For the qRT-PCR analysis of miRNAs, reverse transcription was performed as for mRNA, except that the miRNA was reverse transcribed with Bulge-loop miRNA qRT-PCR Primer Sets (RiboBio, Guangzhou, China). The qRT-PCR process and the data analysis methods were the same as for mRNA. The primers specific to miRNAs were designed by RiboBio. Chicken U6 small nuclear RNA was used as an internal control for normalization of miRNA data.

### PPI Analysis of DEGs

Protein–protein interaction (PPI) analysis of DEGs was based on the STRING database^4^ (Organism: Gallus gallus). We constructed the PPI network by extracting the target gene list from the database pertaining to chickens. In this study, the online tool STRING was applied to analyze the PPI of DEGs, and PPI with scores >700 were selected as significant interactions.

### Statistical Analysis

Statistical analyses of the qRT-PCR results and graphs were carried out in GraphPad Prism (version 5.0) software (San, Diego, CA, United States). Statistical significance of the data was tested by performing paired *t*-tests. The results are presented as means ± SEM of three replicates, and the statistical significance was represented by *p*-values < 0.05, 0.01, or 0.001.

## Results

### The cDNA Library Sequencing and Transcriptome Profiles of the Breast Muscle

**FIGURE 1:**
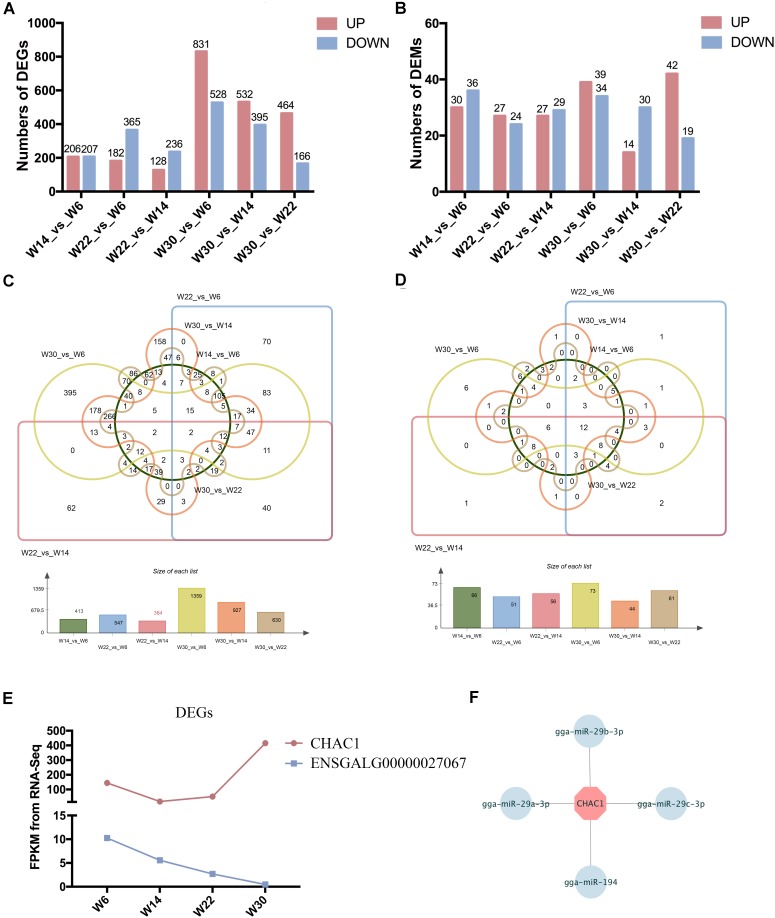
The differentially expressed genes (DEGs) and miRNAs (DEMs) in chicken breast muscle. **(A)** The numbers of DEGs in different comparisons. **(B)** The numbers of DEMs in different comparisons. **(C)** The Wayne diagram of DEGs among the six comparisons. **(D)** The Wayne diagram of DEMs among the six comparisons. **(E)** The FPKM value of two DEGs (*CHAC1* and ENSGALG00000027067) in four developmental stages of chicken breast muscle. **(F)** The miRNA–mRNA pairs of *CHAC1*.

The Illumina Hiseq 2500 platform was used to perform RNA-seq for the 12 cDNA libraries. To preferentially select cDNA fragments 250–300 bp in length, we purified the library fragments with the AMPure XP system (Beckman Coulter, Beverly, MA, United States). The number of raw reads generated from each library exceeded 100 million reads. After filtering of low-quality reads, the clean reads ranged from 81.04 to 105.93 G, and more than 71.33% of the clean reads mapped to the genome (Supplementary Table S2). The GC content of the clean data ranged from 50.24 to 53.87%, and the clean read quality scores of Q20 and Q30 were above 96.48 and 91.06%, respectively, thus demonstrating that the reliability and quality of the sequencing data were adequate for further analysis. Finally, a total of 15,508 known transcripts and 25,718 novel transcripts were identified in the data (Supplementary Table S3). To investigate the key mRNAs involved in chicken breast muscle development, we analyzed the differentially expressed genes (DEGs) at four developmental stages of breast muscle in Gushi chicken. Among the different combinations, the number of up- and down-regulated genes between *W14* vs. *W6*, *W22* vs. *W14*, *W22* vs. *W6*, *W30* vs. *W6*, *W30* vs. *W14*, and *W30* vs. *W22* are shown in Figure 1A, and a list of these DEGs among six comparisons was shown in Supplementary Table S4. The value of the log2-ratio ranged from –9.47317 to 8.00024. The distribution of these DEGs among the different combinations was shown in Figure 1C. Especially, we found that two DEGs comprising *CHAC1* (ENSGALG00000027874) and ENSGALG00000027067 were significantly and differentially expressed in the six combinations (Figure 1E). Thereinto, *CHAC1* was targeted by many miRNAs, such as miR-29a-3p, miR-29b-3p, miR-29c-3p, and miR-194 (Figure 1F).

### Small RNA Library Sequencing and miRNA Transcriptome Profiles of the Breast Muscle

The small RNA libraries were also constructed from the breast muscle tissues collected from chickens in all four growth stages. After filtering of low-quality reads, 11.68 to 13.33 G clean reads were generated, more than 86.29% of which mapped to the genome (Supplementary Table S5). The quality score Q20 and Q30 percentages were above 96.62 and 93.61%, respectively, and the GC content of the clean data ranged from 43.96 to 44.96%. These results demonstrated that the samples were reliable and of high quality. Finally, a total of 388 known miRNAs and 31 novel miRNAs were identified (Supplementary Table S3). To investigate the key miRNAs involved in chicken breast muscle development, we analyzed the DEMs at four developmental stages. Among the different combinations, the number of up- and down-regulated miRNAs in *W14* vs. *W6*, *W22* vs. *W14*, *W22* vs. *W6*, *W30* vs. *W6*, *W30* vs. *W14*, and *W30* vs. *W22* are shown in Figure 1B. Interestingly, twelve DEMs including miR-148a-3p, let-7f-5p, miR-1a-3p, miR-30c-5p, miR-26a-5p, miR-146c-5p, miR-101-3p, let-7i, miR-206, miR-30e-3p, let-7b, and miR-133a-3p, were identified among all six comparisons (Figure 1D).

### Correlation Analysis of Differentially Expressed miRNAs and mRNAs Involved in Breast Muscle Development

**FIGURE 2:**
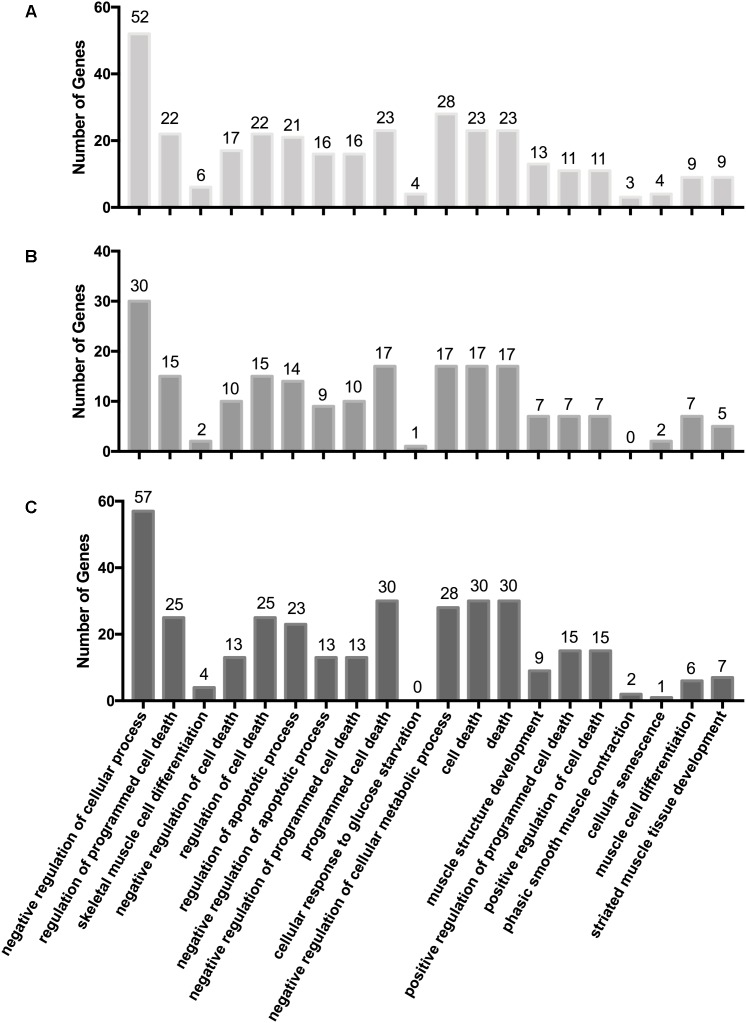
GO terms associated with cellular processes and muscle development. **(A)** *W14* vs. *W6* group. **(B)** *W22* vs. *W14* group. **(C)** *W30* vs. *W22* group.

We integrated the miRNA and mRNA data from Gushi chicken breast muscle, then predicted the potential target genes of DEMs, completed the correlation analysis of DEMs-mRNA pairs, and screened DEMs-mRNA pairs with negatively correlated expression. A total of 795, 714, 494, 2958, 946, and 1053 miRNA–mRNA pairs were predicted of *W14* vs. *W6*, *W22* vs. *W6*, *W22* vs. *W14*, *W30* vs. *W6*, *W30* vs. *W14*, and *W30* vs. *W22*, respectively (Supplementary Table S6). Among these, 417, 370, 240, 1,418, 496, and 363 miRNA–mRNA pairs were found with negatively correlated expression (*q*-value < 0.05). To gain insight into the functions of the miRNA–mRNA pairs with negatively correlated expression between the different developmental stages, we performed GO enrichment analysis to uncover the enriched biological process terms (corrected *p*-values < 0.05) for each comparison. In the *W14* vs. *W6* group, the associations were mainly with negative regulation of biological processes and negative regulation of cellular processes. Moreover, the most significantly enriched GO terms in the *W22* vs. *W6* group were condensed nuclear chromosome outer kinetochore and condensed nuclear chromosome. Enrichment was observed in the GO terms of condensed nuclear chromosome outer kinetochore and condensed nuclear chromosome kinetochore in the *W22* vs. *W14* group. Additionally, in the *W30* vs. *W6* group, terms were related to the extracellular region and extracellular region. However, in the *W30* vs. *W14* group, terms were mainly related to the extracellular region and extracellular space. In the *W30* vs. *W22* group, terms were related to the immune system process and cell surface receptor signaling pathway. In addition, we identified some GO terms related to cellular processes and muscle development in three comparisons (Figure 2). Our results indicated that some GO terms associated with cellular processes in the *W30* vs. *W22* group (Figure 2C) showed the highest value and were followed by that in the *W14* vs. *W6* group (Figure 2A), then the *W22* vs. *W14* group (Figure 2B). However, in almost all muscle development related GO terms, such as skeletal muscle cell differentiation, muscle structure development, phasic smooth muscle contraction and striated muscle tissue development, the highest value was the *W14* vs. *W6* group, followed by the *W30* vs. *W22* group and the *W22* vs. *W14* group. These results indicated that there are differences in the regulation of some biological process related to cellular processes and muscle development in different stages.

### miRNA–mRNA Interaction Network Related to Breast Muscle Development

**FIGURE 3:**
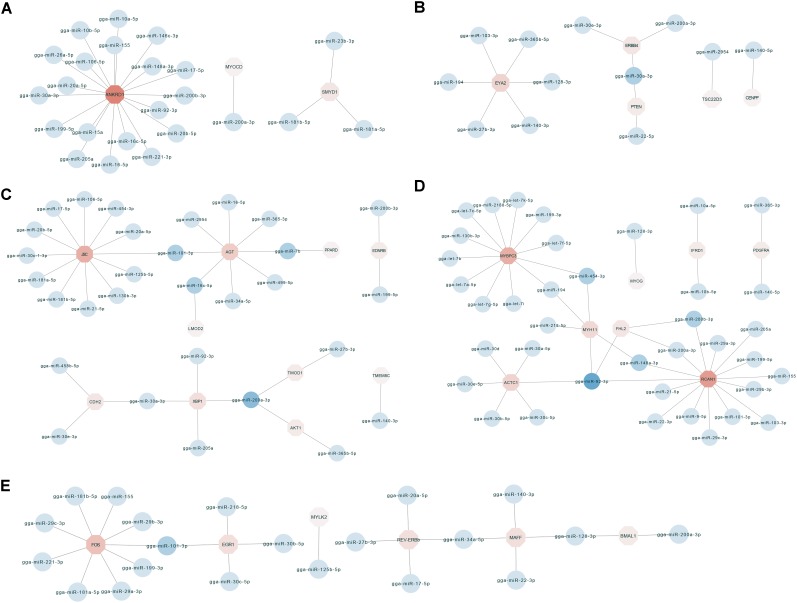
The miRNA–mRNA interaction network related to biological process including muscle cell differentiation, striated muscle tissue development and skeletal muscle cell differentiation. **(A)** The miRNA–mRNA pairs with *ANKRD1* as the core. **(B)** The miRNA–mRNA pairs with *EYA2* as the core. **(C)** The miRNA–mRNA pairs with *JSC* and *AGT* as the core. **(D)** The miRNA–mRNA pairs with *MYBPC3* and *RCAN1* as the core. **(E)** The miRNA–mRNA pairs with *FOS* as the core.

To identify key molecular players in the development of chicken breast muscle, based on the annotations of the negatively correlated miRNA–mRNA pairs in expression level, we focused on some DEG-DEM interactions associated with muscle development (Figure 3). For example, ankyrin repeat domain-containing protein 1 (ANKRD1) was the network core gene (Figure 3A), a total of 23 miRNA–mRNA pairs were found, which were closely related to biological processes such as muscle cell differentiation, striated muscle tissue development and skeletal muscle cell differentiation. Among them, the network core gene *ANKRD1* is targeted by 19 miRNAs, such as miR-10a-5p, miR-17-5p, miR-20a-5p, miR-20b-5p, miR-106a-5p, and miR-30a-3p. Moreover, the network for the important node of eyes absent homolog 2 (EYA2) (Figure 3B) was associated with the biological process of striated muscle tissue development. The results indicated that the *EYA2* is targeted by six miRNAs, such as miR-103-3p and miR-365b-5p, etc. miR-30a-3p targeted phosphatase and tensin homolog deleted on chromosome ten (PTEN), as well as epidermal growth factor receptor (ERBB4). Furthermore, the network focusing on *JSC* and angiotensinogen (AGT) (Figure 3C) was associated with the biological process of muscle cell differentiation. Our results indicated that *JSC* is an important gene targeted by 12 miRNAs, such as miR-101-3p, miR-181a-5p, miR-17-5p, and miR-454-3p, etc. In addition, *AGT* was found to be targeted by miRNAs, including miR-101-3p, miR-499-5p, miR-7b, and miR-16c-5p. miR-200a-3p was found to target X-box binding protein 1 (XBP1), tumor tropomodulin 1 (TMOD1) and *AKT1*. In addition, the networks for kernel of myosin binding protein C (MYBPC3) and regulator of calcineurin 1 (RCAN1) (Figure 3D) were associated with the biological processes of muscle cell differentiation and striated muscle tissue development; myosin heavy chain gene (MYH11), *MYBPC3*, *RCAN1* and alfa cardiac actin (ACTC1) were surrounded by 5, 12, 14, and 6 miRNAs, respectively. For example, *MYH11* was targeted by miRNAs, including miR-454-3p, miR-148a-3p, and miR-194. *MYBPC3* was targeted by let-7b, miR-454-3p, and miR-199-3p. *RCAN1* was targeted by miR-200a-3p, miR-103-3p, and miR-148a-3p. *ACTC1* was targeted by the family of miR-30. Additionally, miR-92-3p targeted *MYH11*, four and a half LIM-only protein 2 (FHL2), *ACTC1* and *RCAN1*. Moreover, in the network with the core node of FOS (Figure 3E), 24 miRNA–mRNA pairs were found, which were associated with the biological processes of skeletal muscle cell differentiation and striated muscle tissue development. Our results showed that *FOS* was targeted by miRNAs including miR-199-3p, miR-181a-5p, and miR-101-3p. Early growth response 1 (EGR1) was targeted by miRNAs including miR-101-3p, miR-30b-5p, and miR-30c-5p. Together, these results show key networks of DEG-DEM interaction during muscle development, thus suggesting that the functions of these common DEGs and DEMs during different developmental stages are mainly involved in breast muscle development.

### miRNA Pathways Related to Breast Muscle Development

**FIGURE 4:**
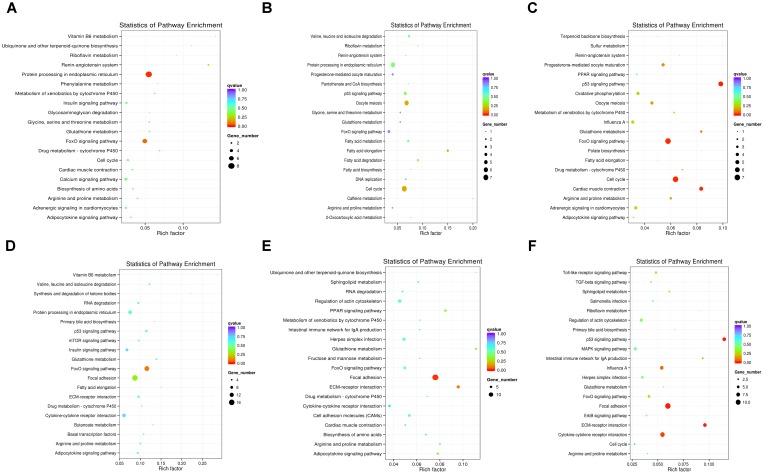
The top 20 KEGG pathways enriched in the six combinations. **(A)** *W14* vs. *W6* group. **(B)** *W22* vs. *W6* group. **(C)** *W22* vs. *W14* group. **(D)** *W30* vs. *W6* group. **(E)** *W30* vs. *W14* group. **(F)** *W30* vs. *W22* group.

To further understand how DEMs work with DEGs in pathways regulating chicken muscle development, we performed KEGG pathway analysis of miRNA–mRNA pairs with negatively correlated expression. The top 20 KEGG terms enriched in the pathway categories for the six combinations are shown in Figure 4. For the *W14* vs. *W6* comparison, the two top pathways were the protein processing in endoplasmic reticulum and the FoxO signaling pathway (Figure 4A). Furthermore, for the *W22* vs. *W6* comparison, the cell cycle pathway and oocyte meiosis pathway were identified as the most significantly enriched pathways (Figure 4B). Additionally, the p53 signaling, cell cycle and FoxO signaling pathways were the top three pathways for the *W22* vs. *W14* comparison (Figure 4C). The FoxO signaling and focal adhesion pathways were the top two pathways for the *W30* vs. *W6* comparison (Figure 4D). In the comparison between the *W30* and *W14* groups, the two top pathways were the focal adhesion and the ECM-receptor interaction pathway (Figure 4E). In addition, for the *W30* vs. *W22* comparison, there were three top pathway terms: p53 signaling, ECM-receptor interaction and focal adhesion pathways (Figure 4F). Interestingly, we found that the FoxO signaling pathway was one of the most frequently enriched pathways. In the *W30* vs. *W6* comparison, 14 DEGs were enriched in the FoxO signaling pathway.

**FIGURE 5:**
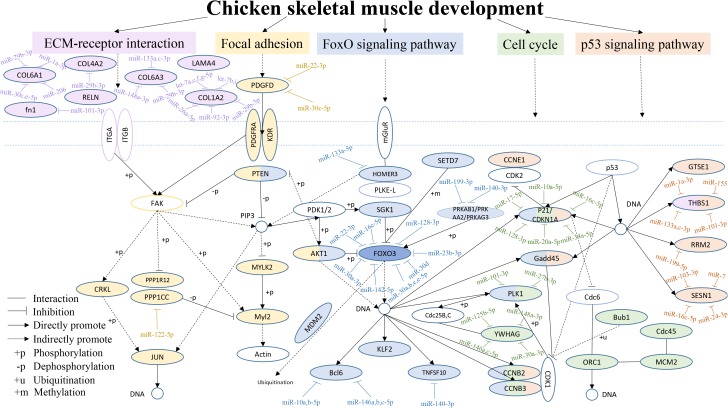
An interaction network of the DEGs and DEMs across five pathways during chicken breast muscle development. The genes within the ECM-receptor interaction, focal adhesion, FoxO signaling, cell cycle and p53 signaling pathways are shown in fuchsia, yellow, purple, green, and orange–red, respectively. The genes along with different pathways are shown in different combined colors. The genes with colors and target miRNAs are differentially expressed genes or miRNAs identified in the study (*p* < 0.05). The genes without color were not in our differentially expressed data and are shown as a supplement.

From the above results, we found that the focal adhesion, ECM-receptor interaction, FoxO signaling, cell cycle and p53 signaling pathways were often among the top 20 KEGG pathways in the six comparisons. Therefore, a hypothetical regulatory network was constructed based on enriched genes in different combinations in the five pathways, to show the interactions of DEGs and DEMs during chicken breast muscle development (Figure 5). This network included key pathways such as the ECM-receptor, focal adhesion, FoxO signaling, cell cycle and p53 signaling pathways, and revealed the cross-talk among these pathways during breast muscle development. The key miRNA–mRNA target networks were also included in this network, which showed that the ECM-receptor pathway was upstream and was followed by the focal adhesion pathway, the FoxO signaling pathway was in the center, and the cell cycle pathway and finally the p53 signaling pathway followed. In addition, in the ECM-receptor pathway, collagen 6A1 (COL6A1), *COL6A3*, *COL1A2*, and *COL4A2* were all targeted by miR-29b-3p. Moreover, the focal adhesion pathway included many genes, such as *JUN*, platelet-derived growth factor alpha receptor (PDGFRA), myosin light chain polypeptide kinase isoform 2 (MYLK2), *PTEN* and v-akt murine thymoma viral oncogene homolog 1 (AKT1). *PTEN* and *AKT1* were important genes that also cross-talk with the FoxO signaling pathway. Interestingly, the FoxO signaling pathway was the central pathway, the Forkhead box O3 (FOXO3) was in the center of the FoxO signaling pathway, and *FOXO3* was targeted by the miR-30 family. Altogether, these results suggested that the FoxO signaling pathway is important for the different stages of muscle development. Furthermore, *PTEN*, *AKT1*, and *HOMER3* were the upstream genes for the FoxO signaling pathway, and HOMRE3 was targeted by miR-133a-5p. Our results showed that polo-like kinase 1 (PLK1) cross-talks with the FoxO signaling pathway and cell cycle pathway, and it is targeted by miR-101-3p and miR-27b-3p. GADD45 is part of the FoxO and p53 signaling pathways. Moreover, *P21*, Cyclin B2 (CCNB2) and Cyclin B3 (CCNB3) cross-talk with the FoxO signaling, cell cycle and p53 signaling pathways, and *P21* is an important gene targeted by miRNAs including miR-10a-5p, miR-17-5p, and miR-20a-5p. In the p53 signaling pathway, thrombospondin-1 (THBS1) was also found to cross-talk with the ECM-receptor interaction pathway and to be the important gene in this pathway. THBS1 is targeted by the miR-1a-3p, miR-133a-3p, and miR-101-3p. Moreover, cell division cycle 45-cell (CDC45), origin recognition complexes 1 (ORC1), minichromosome maintenance 2 (MCM2) and budding uninhibited by benzimid Azole1 (BUB1) belong to the cell cycle pathway. Together, these results revealed that the above five pathways and their DEG–DEM interactions play critical roles in chicken breast muscle development.

### PPI Network Related to Breast Muscle Development

**FIGURE 6:**
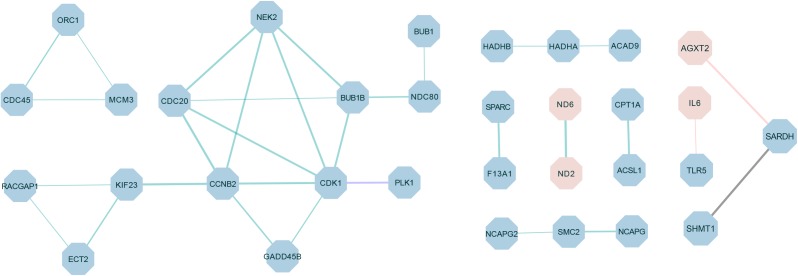
The protein–protein interaction (PPI) network of differentially expressed proteins in different comparisons. The green connection represents *W22* vs. *W6*, the purple connection represents *W22* vs. *W14*, the red connection represents *W30* vs. *W6* and the black connection represents *W30* vs. *W14*. Red represents up-regulated genes, and blue represents down-regulated genes. The thickness of the lines indicates the level of confidence in protein interactions.

The PPI network was constructed by using the extracted target gene list from the STRING database in Cytoscape (Figure 6). The PPI network from DEGs of the *W22* vs. *W6* comparison contained 27 protein–protein pairs. Thereinto, ND6-ND2 were up-regulated, and the other 26 pairs such as *CDK1-PLK1*, *CDC45-ORC1*, *MCM3-ORC1*, *CDC45-MCM3*, *CCNB2-CDK1*, *GADD45B-CCNB2*, *GADD45B-CDK1*, and *CDK1-BUB1B* were down-regulated. Moreover, the *W22* vs. *W14* comparison included one protein–protein pair, *PLK1-CDK1*, of down-regulated genes. Furthermore, the DEGs from the *W30* vs. *W6* comparison included two protein–protein pairs, *IL6-TLR5* and *AGXT2-SARDH*. Interleukin-6 (IL6) and alanine-glyoxylate aminotransferase 2 (AGXT2) were up-regulated genes, and Toll-like Receptor 5 (TLR5) and serine hydroxymethyltransferase 1 (SHMT1) were down-regulated genes. In addition, the DEGs from the *W30* vs. *W14* comparison included one protein–protein pair, *SARDH-SHMT1*, of down-regulated genes.

### Verification of miRNA and mRNA Expression Profiles With qRT-PCR

**FIGURE 7:**
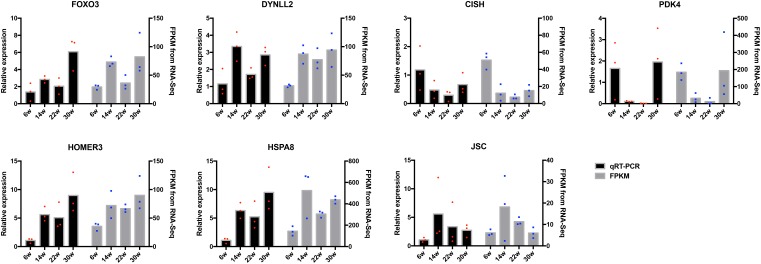
Quantitative real-time PCR (qRT-PCR) validation of differentially expressed mRNAs. The red dots indicate the biological repetition in qRT-PCR result. The blue dots indicate the biological repetition from RNA-seq.

**FIGURE 8:**
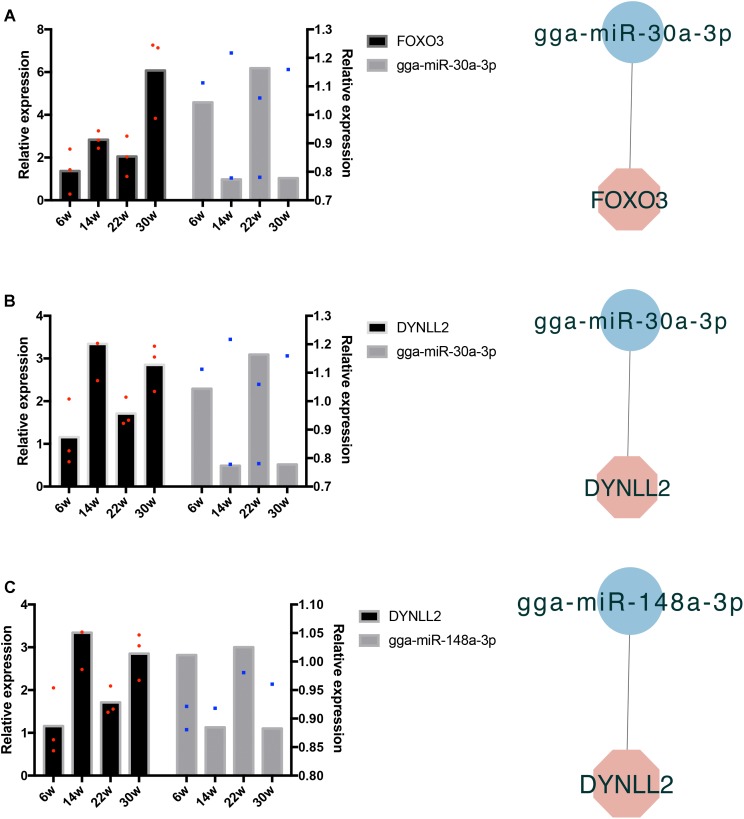
Quantitative real-time PCR validation of differentially expressed miRNAs and their corresponding target mRNAs. The red dots indicate three biological repetition of gene in qRT-PCR result. The blue dots indicate three biological repetition of miRNA in qRT-PCR result. **(A)** The qRT-PCR validation of miR-30a-3p and its target gene *FOXO3*; **(B)** The qRT-PCR validation of miR-30a-3p and its target gene DYNLL2; **(C)** The qRT-PCR validation of miR-148a-3p and its target gene DYNLL2.

The frequently occurring genes in the miRNA–mRNA pairs, including *FOXO3*, dynein light chain (DYNLL2), chromogenic *in situ* hybridization (CISH), *PDK4*, *HOMER3*, heat shock protein alpha 8 (HSPA8), and *JSC*, were selected for qRT-PCR validation. The gene expression levels were determined and compared with the RNA-seq data, which showed similar patterns in comparison to the RNA-seq data (Figure 7), thus suggesting that the RNA-seq data were credible. Furthermore, three miRNA–mRNA connections were negatively correlated, miR-30a-3p-FOXO3, miR-30a-3p-DYNLL2, and miR-148a-3p-DYNLL2, thus indicating that they have potential interactivity (Figure 8).

## Discussion

Muscle development is the result of precise regulation of hormones, genes, non-coding RNAs and other factors, such as spatiotemporal expression, signal cascade, transcriptional regulation and feedback mechanisms (Nakasa et al., 2010; Moncaut et al., 2013; Huang et al., 2018). Over the past few years, many studies have been performed on gene identification and functional research, miRNA identification and pathway regulation regarding chicken skeletal muscle development. However, these studies have focused on embryonic and early post-emergence (Li et al., 2016; Luo et al., 2016). There is still a lack of understanding of the molecular mechanisms underlying skeletal muscle development in the late stage of chicken hatching. Therefore, it is important to determine the molecular mechanisms underlying the postnatal development of chicken skeletal muscle. In this research, we performed RNA-seq to assess four representative developmental stages (6, 14, 22, and 30 weeks) of Gushi chicken breast muscle after birth, then constructed miRNA and mRNA profiles related to breast muscle development. A total of 15,508 known transcripts, 25,718 novel transcripts, 388 known miRNAs and 31 novel miRNAs were identified in the breast muscle samples (Supplementary Table S3). These data provide a valuable resource for analysis of the molecular mechanisms underlying the late development of chicken skeletal muscle and post-transcriptional regulation.

The Gushi chicken is a Chinese local breed used for eggs and meat. The flesh is tender and uniquely flavored, and this breed has important development and utilization value. Therefore, understanding the molecular mechanisms underlying Gushi chicken muscle development is important for this breed’s development and utilization. Compared with other varieties, Gushi chicken grows slowly. However, our past research has indicated that the first 22 weeks is an important stage in the development of breast muscle in Gushi chicken. Before 22 weeks, there is a significant negative correlation between breast muscle fiber diameter and density. During this period, the diameter of breast muscle fibers increases rapidly, and the density decreases sharply. After 22 weeks, the growth of breast muscle fiber diameter and density is relatively balanced (Fu et al., 2018). At present, there is a lack of understanding of the molecular mechanisms underlying the developmental characteristics of Gushi chicken breast muscle. Differentially expressed genes and miRNAs may have important regulatory roles in muscle development. In this study, 2 DEGs (*CHAC1* and ENSGALG00000027067) and 12 DEMs (miR-148a-3p, let-7f-5p, miR-1a-3p, miR-30c-5p, miR-26a-5p, miR-146c-5p, miR-101-3p, let-7i, miR-206, miR-30e-3p, let-7b, and miR-133a-3p) were identified as differentially expressed among all six comparisons. Thereinto, it showed that the *CHAC1* is involved in growth of fast skeletal muscle in Atlantic salmon (Bower and Johnston, 2010). Moreover, we screened DEMs-mRNA pairs at four developmental stages in Gushi chicken breast muscle and then performed functional enrichment analysis. The KEGG pathway enrichment results showed that in the *W22* vs. *W14* comparison, the genes were mostly enriched in pathways involved in muscle development. These results indicate that 14–22 weeks is an important stage in the development of Gushi chicken breast muscle, in agreement with findings from our previous histological analysis (Fu et al., 2018). Therefore, the miRNA and mRNA expression profiles obtained in this study reflect the late developmental regulation of Gushi chicken breast muscle at the molecular level.

Skeletal muscle development involves many biological processes. To gain insight into how miRNAs and their target genes regulate muscle development, we constructed the interaction networks between miRNAs and mRNAs related to muscle development (Figure 3). Among these interaction networks, *ANKRD1* was predicted to be a target of miRNAs including miR-30a-3p, miR-17-5p, miR-20a-5p, miR-20b-5p, and miR-106a-5p. This gene is also involved in the biological processes of skeletal muscle cell differentiation, muscle structure development, muscle cell differentiation and striated muscle tissue development. *ANKRD1* plays a regulatory role in smooth muscle (Mohamed et al., 2011). miR-30a-3p has also been suggested to regulate muscle differentiation (Zhang et al., 2016). miR-17-5p, miR-20a, miR-106a and miR-20b are members of the miR-17 family, which play important roles in cellular differentiation and early mammalian development (Foshay and Gallicano, 2009; Luo et al., 2016). In addition, among these networks, the most complex miRNA–mRNA interaction network was based on *MYBPC3* and *RCAN1* (Figure 3D). Furthermore, the network is related to muscle cell differentiation and striated muscle tissue development. The interaction network consists of eight core genes including *MYBPC3*, *MYH11*, α-actin gene (ACTC1), *FHL2* and *RCAN1*, and 38 miRNAs such as miR-454-3p and miR-92-3p, etc. Some studies have confirmed that *MYBPC* is essential for the sarcomere structure of striated muscle (Tajsharghi et al., 2010). A variant in *MYH11*, *R247C*, alters the contractile function of myosin (Kuang et al., 2012). Mutations in *ACTC1* cause muscle diseases in the early postnatal period (Boutilier et al., 2017). *FHL2* has roles in post-injury inflammation or cytoprotection of muscle cells (Wong et al., 2012). In addition, over-expression of miR-454-3p shifts the cell cycle arrest from G2/M phase to S phase (Wu et al., 2014). Moreover, the miRNA–mRNA interaction networks of the core genes of *EYA2* (Figure 3B), *JSC* and *AGT* (Figure 3C), and *FOS* and *EGR1* (Figure 3E), are closely related to the biological processes of striated muscle tissue development, muscle cell differentiation and skeletal muscle cell differentiation. These findings suggest that these miRNA–mRNA interaction networks may play a key role in the late developmental regulation of Gushi chicken breast muscle, thus providing new insights for further understanding of the regulation mechanisms of the skeletal muscle development of chicken.

Postnatal growth of skeletal muscle is mainly achieved by increasing the hypertrophy of existing muscle fibers (Jiang et al., 2010). Therefore, the pathways involved in adhesion, growth, migration and differentiation of muscle cells might be critical for chicken growth. In our study, the ECM-receptor interaction, focal adhesion, FoxO signaling, cell cycle and p53 signaling pathways were found at a high frequency in the different development stage of Gushi chicken breast muscle after birth (Figure 4). Similar results have been reported in previous studies (Chen et al., 2015; Xue et al., 2017). In fact, the extracellular matrix (ECM) plays an integral role in many cellular responses such as transcription and inflammation, migration, proliferation and differentiation (Freedman et al., 2018). Focal adhesions have been considered as mechanical linkages to the ECM and are the signaling centers of numerous intracellular pathways involved in cell motility, proliferation and differentiation (Romer et al., 2006). In addition, the FoxO signaling pathway is known to play a key role in cell cycle regulation (Ren et al., 2018), thus implicating enrichment pathways related to skeletal muscle growth and development. Therefore, ECM-receptor interaction, focal adhesion, FoxO signaling, cell cycle and p53 signaling pathways may be keys to further explore the molecular mechanisms underlying chicken skeletal muscle development regulation.

To gain insight into the interactions among chicken breast muscle developmental genes, miRNAs and pathways, we determined the interaction networks of five key pathways (ECM-receptor interaction, focal adhesion, FoxO signaling, cell cycle and p53 signaling pathways), on the basis of Gushi chicken breast muscle development-related miRNA and transcriptome data (Figure 5). We found that the FoxO signaling pathway resides in the center of other signaling pathways, and *FOXO3* is located at the center of FoxO signaling pathway. *FOXO3* is associated with many genes and targeted by several miRNAs including the miR-30 family. *FOXO3* is a candidate gene known to be involved in chicken growth (Chen et al., 2015; Xue et al., 2017). Many *FOXO3* targets play regulatory roles in myoblast proliferation and differentiation and muscle fiber hypertrophy. The miR-30 family microRNAs are related to endoplasmic reticulum stress in cardiac muscle and vascular smooth muscle cells (Chen et al., 2014). These findings indicate that *FOXO3* may be a critical gene involved in muscle development in postnatal growth. *FOXO3* affects muscle development, perhaps through regulation by upstream genes such as *AKT1*, *SKG1*, *SETD7*, and *PRKAB1*, and downstream genes such as *KLF2*, *BCL6*, and *TNFSF10*, as well as miRNAs. Moreover, the FoxO signaling pathway is linked to the focal adhesion, p53 signaling and cell cycle pathways via core genes such as *PTEN*, *AKT1*, *GADD45*, *PLK1*, *CCNB2*, and *CCNB3* (Figure 5), and it plays a regulatory role in biological processes such as muscle cell differentiation and striated muscle tissue development. The *AKT* protein kinase signaling pathway controls the cell survival and proliferation of invertebrate organisms in mammals by inhibiting the activity of members of the FOXO transcription factor family (Tran et al., 2002). Furthermore, the changes in PTEN/AKT signaling in vascular smooth muscle cells after vascular injury are associated with cell proliferation (Smith et al., 2011). These results indicate that the key core node genes are important links among pathways and between genes and miRNAs within pathways. This interaction network of pathways provides a new understanding of the molecular regulation mechanisms underlying chicken skeletal muscle development.

Moreover, we also demonstrated the interactions among genes in the development of Gushi chicken breast muscle, such as the PPI network of Polo-Like Kinase 1 (PLK1) and *CDK1* (Figure 6). *CDK1* and *PLK1* are regulatory elements that control centrosome separation. In G2 phase, *PLK1* can be isolated independently of the *CDK1* trigger centrosome (Smith et al., 2011). In addition, *PLK1* is linked to the FoxO signaling and cell cycle pathways (Figure 5), which are involved in cell cycle regulation. Among the PPI network of *CCNB2*, *CDK1*, and *GADD45B* (Figure 6), the DNA damage response gene *GADD45a* appears to be a direct target of *FOXO3a*, and it mediates part of the role of *FOXO3a* in DNA repair (Tran et al., 2002). In our data, these three interacting genes were differentially expressed during the development of Gushi chicken breast muscle, and the same expression trend has also been observed in MMQ tumor stem-like cells (Su et al., 2017). Moreover, *CDC45*, *ORC1*, *MCM3*, and BUB1 belong to the cell cycle pathway, and we determined an interaction between *BUB1* and *CDK1*, as well as among *CDC45*, *ORC1*, and *MCM3* (Figure 6). Studies have shown that Cdk1-mediated phosphorylation of *BUBR1* controls checkpoint arrest and chromosome segregation (Wong and Fang, 2007). Another study also indicated that *CDC45* interacts with *ORC1* and *ORC6*, and weakly interacts with *MCM3* (Kneissl et al., 2003). Thus, the interactions among genes in these pathways affect muscle development, and the interactions of miRNAs and mRNAs in these pathways may provide new insights into the regulation of breast muscle development in chickens. The above results indicate that the presence of complex inter-gene interactions in the development of Gushi chicken breast muscle.

In summary, we described the miRNA and transcriptome profiles of Gushi chicken breast muscle at the developmental stages of 6, 14, 22, and 30 weeks, and we constructed the interaction network among proteins, miRNAs and mRNAs and signaling pathways involved in breast muscle development. These results provide a valuable resource for further study of the molecular mechanism underlying late stages of chicken skeletal muscle development, as well as new insights for better understanding of the interactions among various factors related to chicken skeletal muscle development and regulation. Further research should validate the functions of these key interaction networks and their components in chicken skeletal muscle development at the cellular level.

## Data Availability

All the raw sequences from this study have been deposited in the NCBI database Sequence Read Archive with the accession number PRJNA516810 (BioProject ID of mRNA) and PRJNA516961 (BioProject ID of miRNA).

## Author Contributions

YL performed research, analyzed data, and wrote the manuscript. YC, WJ, and SF analyzed data and was involved in the study design. DL and YZ performed the statistical analysis. GS, RJ, RH, ZL, and XK were involved in the design of the study. GL conceived the study and involved in its design and coordination. All authors contributed to manuscript revision, read and approved the submitted version.

## Conflict of Interest Statement

The authors declare that the research was conducted in the absence of any commercial or financial relationships that could be construed as a potential conflict of interest.

## Footnotes

1 https://sourceforge.net/projects/bowtie-bio/files/

2 http://www.geneontology.org/

3 http://www.genome.jp/kegg/

4 https://string-db.org

## References

[B1] Alexander M. S. Kawahara G. Motohashi N. Casar J. C. Eisenberg I. Myers J. A. (2013). MicroRNA-199a is induced in dystrophic muscle and affects WNT signaling, cell proliferation, and myogenic differentiation. *Cell Death Differ.* 20 1194–1208. 10.1038/cdd.2013.62 23764775 PMC3741500

[B2] Badodi S. Baruffaldi F. Ganassi M. Battini R. Molinari S. (2015). Phosphorylation-dependent degradation of MEF2C contributes to regulate G2/M transition. *Cell Cycle* 14 1517–1528. 10.1080/15384101.2015.1026519 25789873 PMC4615021

[B3] Boutilier J. K. Taylor R. L. Ram R. McNamara E. Nguyen Q. Goullee H. (2017). Variable cardiac alpha-actin (Actc1) expression in early adult skeletal muscle correlates with promoter methylation. *Biochim. Biophys. Acta Gene Regul. Mech.* 1860 1025–1036. 10.1016/j.bbagrm.2017.08.004 28847732

[B4] Bower N. I. Johnston I. A. (2010). Discovery and characterization of nutritionally regulated genes associated with muscle growth in Atlantic salmon. *Physiol. Genom.* 42A, 114–130. 10.1152/physiolgenomics.00065.2010 20663983 PMC2957792

[B5] Chen B. Xu J. He X. Xu H. Li G. Du H. (2015). A genome-wide mRNA screen and functional analysis reveal FOXO3 as a candidate gene for chicken growth. *PLoS One* 10:e0137087. 10.1371/journal.pone.0137087 26366565 PMC4569328

[B6] Chen M. Ma G. Yue Y. Wei Y. Li Q. Tong Z. (2014). Downregulation of the miR-30 family microRNAs contributes to endoplasmic reticulum stress in cardiac muscle and vascular smooth muscle cells. *Int. J. Cardiol.* 173 65–73. 10.1016/j.ijcard.2014.02.007 24612558

[B7] Enright A. J. John B. Gaul U. Tuschl T. Sander C. Marks D. S. (2003). MicroRNA targets in *Drosophila*. *Genome Biol.* 5:R1. 10.1186/gb-2003-5-1-r1 14709173 PMC395733

[B8] Foshay K. M. Gallicano G. I. (2009). miR-17 family miRNAs are expressed during early mammalian development and regulate stem cell differentiation. *Dev. Biol.* 326 431–443. 10.1016/j.ydbio.2008.11.016 19073166

[B9] Freedman B. R. Rodriguez A. B. Leiphart R. J. Newton J. B. Ban E. Sarver J. J. (2018). Dynamic loading and tendon healing affect multiscale tendon properties and ECM stress transmission. *Sci. Rep.* 8:10854. 10.1038/s41598-018-29060-y 30022076 PMC6052000

[B10] Friedlander M. R. Mackowiak S. D. Li N. Chen W. Rajewsky N. (2012). miRDeep2 accurately identifies known and hundreds of novel microRNA genes in seven animal clades. *Nucleic Acids Res.* 40 37–52. 10.1093/nar/gkr688 21911355 PMC3245920

[B11] Fu S. Zhao Y. Li Y. Li G. Chen Y. Li Z. (2018). Characterization of mirna transcriptome profiles related to breast muscle development and intramuscular fat deposition in chickens. *J. Cell Biochem.* 119 7063–7079. 10.1002/jcb.27024 29737555

[B12] Gagan J. Dey B. K. Layer R. Yan Z. Dutta A. (2012). Notch3 and Mef2c proteins are mutually antagonistic via Mkp1 protein and miR-1/206 microRNAs in differentiating myoblasts. *J. Biol. Chem.* 287 40360–40370. 10.1074/jbc.M112.378414 23055528 PMC3504751

[B13] Hamarneh S. R. Murphy C. A. Shih C. W. Frontera W. Torriani M. Irazoqui J. E. (2015). Relationship between serum IGF-1 and skeletal muscle IGF-1 mRNA expression to phosphocreatine recovery after exercise in obese men with reduced GH. *J. Clin. Endocrinol. Metab.* 100 617–625. 10.1210/jc.2014-2711 25375982 PMC4318910

[B14] Huang Y. Wen H. Zhang M. Hu N. Si Y. Li S. (2018). The DNA methylation status of MyoD and IGF-I genes are correlated with muscle growth during different developmental stages of Japanese flounder (Paralichthys olivaceus). *Comp. Biochem. Physiol. B. Biochem. Mol. Biol.* 219–220 33–43. 10.1016/j.cbpb.2018.02.005 29486246

[B15] Jebessa E. Ouyang H. Abdalla B. A. Li Z. Abdullahi A. Y. Liu Q. (2018). Characterization of miRNA and their target gene during chicken embryo skeletal muscle development. *Oncotarget* 9 17309–17324. 10.18632/oncotarget.22457 29707110 PMC5915118

[B16] Jiang C. Shi P. Li S. Dong R. Tian J. Wei J. (2010). Gene expression profiling of skeletal muscle of nursing piglets. *Int. J. Biol. Sci.* 6 627–638. 10.7150/ijbs.6.627 20975821 PMC2962265

[B17] Kneissl M. Putter V. Szalay A. A. Grummt F. (2003). Interaction and assembly of murine pre-replicative complex proteins in yeast and mouse cells. *J. Mol. Biol.* 327 111–128. 10.1016/S0022-2836(03)00079-2 12614612

[B18] Kuang S. Q. Kwartler C. S. Byanova K. L. Pham J. Gong L. Prakash S. K. (2012). Rare, nonsynonymous variant in the smooth muscle-specific isoform of myosin heavy chain, MYH11, R247C, alters force generation in the aorta and phenotype of smooth muscle cells. *Circ. Res.* 110 1411–1422. 10.1161/CIRCRESAHA.111.261743 22511748 PMC3917690

[B19] Li G. Song Y. Li G. Ren J. Xie J. Zhang Y. (2018). Downregulation of microRNA21 expression inhibits proliferation, and induces G1 arrest and apoptosis via the PTEN/AKT pathway in SKM1 cells. *Mol. Med. Rep.* 18 2771–2779. 10.3892/mmr.2018.9255 30015844 PMC6102657

[B20] Li Z. Ouyang H. Zheng M. Cai B. Han P. Abdalla B. A. (2016). Integrated analysis of long non-coding RNAs (LncRNAs) and mRNA expression profiles reveals the potential role of LncRNAs in skeletal muscle development of the chicken. *Front. Physiol.* 7:687. 10.3389/fphys.2016.00687 28119630 PMC5220077

[B21] Luo W. Lin S. Li G. Nie Q. Zhang X. (2016). Integrative analyses of miRNA-mRNA interactions reveal let-7b, miR-128 and MAPK pathway involvement in muscle mass loss in sex-linked dwarf chickens. *Int. J. Mol. Sci.* 17:276. 10.3390/ijms17030276 26927061 PMC4813140

[B22] Luo W. Wu H. Ye Y. Li Z. Hao S. Kong L. (2014). The transient expression of miR-203 and its inhibiting effects on skeletal muscle cell proliferation and differentiation. *Cell Death Dis.* 5:e1347. 10.1038/cddis.2014.289 25032870 PMC4123083

[B23] Ma M. Z. Li C. X. Zhang Y. Weng M. Z. Zhang M. D. Qin Y. Y. (2014). Long non-coding RNA HOTAIR, a c-Myc activated driver of malignancy, negatively regulates miRNA-130a in gallbladder cancer. *Mol. Cancer* 13:156. 10.1186/1476-4598-13-156 24953832 PMC4085645

[B24] Mancinelli R. Pietrangelo T. Burnstock G. Fano G. Fulle S. (2012). Transcriptional profile of GTP-mediated differentiation of C2C12 skeletal muscle cells. *Purinergic. Signal.* 8 207–221. 10.1007/s11302-011-9266-3 22127439 PMC3350577

[B25] Mao X. Cai T. Olyarchuk J. G. Wei L. (2005). Automated genome annotation and pathway identification using the KEGG Orthology (KO) as a controlled vocabulary. *Bioinformatics* 21 3787–3793. 10.1093/bioinformatics/bti430 15817693

[B26] Mohamed J. S. Hajira A. Li Z. Paulin D. Boriek A. M. (2011). Desmin regulates airway smooth muscle hypertrophy through early growth-responsive protein-1 and microRNA-26a. *J. Biol. Chem.* 286 43394–43404. 10.1074/jbc.M111.235127 21903578 PMC3234798

[B27] Mok G. F. Lozano-Velasco E. Maniou E. Viaut C. Moxon S. Wheeler G. (2018). miR-133-mediated regulation of the Hedgehog pathway orchestrates embryo myogenesis. *Development* 145:dev.159657. 10.1242/dev.159657 29802149 PMC6031409

[B28] Moncaut N. Rigby P. W. Carvajal J. J. (2013). Dial M(RF) for myogenesis. *FEBS J.* 280 3980–3990. 10.1111/febs.12379 23751110

[B29] Nakasa T. Ishikawa M. Shi M. Shibuya H. Adachi N. Ochi M. (2010). Acceleration of muscle regeneration by local injection of muscle-specific microRNAs in rat skeletal muscle injury model. *J. Cell. Mol. Med.* 14 2495–2505. 10.1111/j.1582-4934.2009.00898.x 19754672 PMC3823166

[B30] Qadir A. S. Woo K. M. Ryoo H. M. Yi T. Song S. U. Baek J. H. (2014). MiR-124 inhibits myogenic differentiation of mesenchymal stem cells via targeting Dlx5. *J. Cell. Biochem.* 115 1572–1581. 10.1002/jcb.24821 24733577

[B31] Ren L. Zhao Y. Huo X. Wu X. (2018). MiR-155-5p promotes fibroblast cell proliferation and inhibits FOXO signaling pathway in vulvar lichen sclerosis by targeting FOXO3 and CDKN1B. *Gene* 653 43–50. 10.1016/j.gene.2018.01.049 29339071

[B32] Romer L. H. Birukov K. G. Garcia J. G. (2006). Focal adhesions: paradigm for a signaling nexus. *Circ. Res.* 98 606–616. 10.1161/01.RES.0000207408.31270.db 16543511

[B33] Schreiber M. Kolbus A. Piu F. Szabowski A. Mohle-Steinlein U. Tian J. (1999). Control of cell cycle progression by c-Jun is p53 dependent. *Genes Dev.* 13 607–619. 10.1101/gad.13.5.607 10072388 PMC316508

[B34] Smith E. Hegarat N. Vesely C. Roseboom I. Larch C. Streicher H. (2011). Differential control of Eg5-dependent centrosome separation by Plk1 and Cdk1. *EMBO J.* 30 2233–2245. 10.1038/emboj.2011.120 21522128 PMC3117641

[B35] Su Z. Cai L. Lu J. Li C. Gui S. Liu C. (2017). Global expression profile of tumor stem-like cells isolated from MMQ rat prolactinoma cell. *Cancer Cell Int.* 17:15. 10.1186/s12935-017-0390-1 28163656 PMC5282624

[B36] Sun Y. Ge Y. Drnevich J. Zhao Y. Band M. Chen J. (2010). Mammalian target of rapamycin regulates miRNA-1 and follistatin in skeletal myogenesis. *J. Cell Biol.* 189 1157–1169. 10.1083/jcb.200912093 20566686 PMC2894448

[B37] Tajsharghi H. Leren T. P. Abdul-Hussein S. Tulinius M. Brunvand L. Dahl H. M. (2010). Unexpected myopathy associated with a mutation in MYBPC3 and misplacement of the cardiac myosin binding protein C. *J. Med. Genet.* 47 575–577. 10.1136/jmg.2009.072710 19858127

[B38] Tran H. Brunet A. Grenier J. M. Datta S. R. Fornace A. J. DiStefano P. S. (2002). DNA repair pathway stimulated by the forkhead transcription factor FOXO3a through the Gadd45 protein. *Science* 296 530–534. 10.1126/science.1068712 11964479

[B39] Trapnell C. Salzberg S. L. (2009). How to map billions of short reads onto genomes. *Nat. Biotechnol.* 27 455–457. 10.1038/nbt0509-455 19430453 PMC2836519

[B40] Wen M. Shen Y. Shi S. Tang T. (2012). miREvo: an integrative microRNA evolutionary analysis platform for next-generation sequencing experiments. *BMC Bioinformatics* 13:140. 10.1186/1471-2105-13-140 22720726 PMC3410788

[B41] Wong C. H. Mak G. W. Li M. S. Tsui S. K. (2012). The LIM-only protein FHL2 regulates interleukin-6 expression through p38 MAPK mediated NF-kappaB pathway in muscle cells. *Cytokine* 59 286–293. 10.1016/j.cyto.2012.04.044 22633286

[B42] Wong O. K. Fang G. (2007). Cdk1 phosphorylation of BubR1 controls spindle checkpoint arrest and Plk1-mediated formation of the 3F3/2 epitope. *J. Cell Biol.* 179 611–617. 10.1083/jcb.200708044 17998400 PMC2080899

[B43] Wu H. J. Ma Y. K. Chen T. Wang M. Wang X. J. (2012). PsRobot: a web-based plant small RNA meta-analysis toolbox. *Nucleic Acids Res.* 40 W22–W28. 10.1093/nar/gks554 22693224 PMC3394341

[B44] Wu X. Ding N. Hu W. He J. Xu S. Pei H. (2014). Down-regulation of BTG1 by miR-454-3p enhances cellular radiosensitivity in renal carcinoma cells. *Radiat. Oncol.* 9:179. 10.1186/1748-717X-9-179 25115181 PMC4252025

[B45] Xue Q. Zhang G. Li T. Ling J. Zhang X. Wang J. (2017). Transcriptomic profile of leg muscle during early growth in chicken. *PLoS One* 12:e0173824. 10.1371/journal.pone.0173824 28291821 PMC5349469

[B46] Young M. D. Wakefield M. J. Smyth G. K. Oshlack A. (2010). Gene ontology analysis for RNA-seq: accounting for selection bias. *Genome Biol.* 11:R14. 10.1186/gb-2010-11-2-r14 20132535 PMC2872874

[B47] Yu M. Wang H. Xu Y. Yu D. Li D. Liu X. (2015). Insulin-like growth factor-1 (IGF-1) promotes myoblast proliferation and skeletal muscle growth of embryonic chickens via the PI3K/Akt signalling pathway. *Cell Biol. Int.* 39 910–922. 10.1002/cbin.10466 25808997

[B48] Zetser A. Gredinger E. Bengal E. (1999). p38 mitogen-activated protein kinase pathway promotes skeletal muscle differentiation. Participation of the Mef2c transcription factor. *J. Biol. Chem.* 274 5193–5200. 10.1074/jbc.274.8.5193 9988769

[B49] Zhang B. W. Cai H. F. Wei X. F. Sun J. J. Lan X. Y. Lei C. Z. (2016). miR-30-5p regulates muscle differentiation and alternative splicing of muscle-related genes by targeting MBNL. *Int. J. Mol. Sci.* 17:182. 10.3390/ijms17020182 26840300 PMC4783916

