# Supplementary material for: Analyses of MicroRNA and mRNA Expression Profiles Reveal the Crucial Interaction Networks and Pathways for Regulation of Chicken Breast Muscle Development
Source: Front Genet. 2019 Mar 18;10:197. doi: 10.3389/fgene.2019.00197 (PMC6431651; doi:10.3389/fgene.2019.00197)
Supplement: Supplementary file 1 [file Table_1.docx]

**Table S1. The qRT-PCR primers used in this study.**

| **Gene** | **Primer** | **Sequence（5’-3’）** | **GenBank no.** | **AT(**°**C)** |
| --- | --- | --- | --- | --- |
| *CHAC1* | F | CCTGCAAGAAGTTCTCCCTTT | NM_001199656.1 | 60 |
|  | R | CCTGCAAGAAGTTCTCCCTTT |  |  |
| *CISH* | F | TCCCGCAGATAGGAGAAGGTC | NM_204626.1 | 60 |
|  | R | TCCCGCAGATAGGAGAAGGTC |  |  |
| *DYNLL2* | F | TGCAACCTGACCCAAGTAAAA | NM_001302156.1 | 60 |
|  | R | TGCAACCTGACCCAAGTAAAA |  |  |
| *FOXO3* | F | TACCCAACTCTGTGATGAAGC | XM_001234495.5 | 60 |
|  | R | AAATTCCCAAGTGACTTAGACC |  |  |
| *HOMER3* | F | TGGTGAGATGTGATACTGAGGG | XM_418233.6 | 60 |
|  | R | TGTATGGATTGGGCTTTGCTT |  |  |
| *HSPA8* | F | ATCTTCTTCAGACGCTCCTTC | NM_205003.2 | 60 |
|  | R | AATCCTGCCCTCAGTATCACA |  |  |
| *JSC* | F | AATCCTGCCCTCAGTATCACA | AF285876.2 | 60 |
|  | R | AGCCTTGCTTCTGCTGGTCAT |  |  |
| *PDK4* | F | AGCCTTGCTTCTGCTGGTCAT | NM_001199909.1 | 60 |
|  | R | TGCACTGAAGGGGTGCTTAG |  |  |

Note: AT, annealing temperature; F, forward primer; R, reverse primer.
