# Supplementary material for: Analyses of MicroRNA and mRNA Expression Profiles Reveal the Crucial Interaction Networks and Pathways for Regulation of Chicken Breast Muscle Development
Source: Front Genet. 2019 Mar 18;10:197. doi: 10.3389/fgene.2019.00197 (PMC6431651; doi:10.3389/fgene.2019.00197)
Supplement: Supplementary file 2 [file Table_2.docx]

**Table S2. Statistics for the cDNA RNA library sequences of Gushi chicken breast muscle.**

| **Library name** | **Clean reads** | **Mapped reads** | **Q20(%)** | **Q30(%)** | **GC content (%)** |
| --- | --- | --- | --- | --- | --- |
| W6_1 | 101,782,844 | 83,373,496 (81.91%) | 97.79 | 94.25 | 50.53 |
| W6_2 | 94,051,690 | 75,121,361 (79.87%) | 97.56 | 93.69 | 51.85 |
| W6_3 | 95,671,700 | 76,160,188 (79.61%) | 97.65 | 93.86 | 52.30 |
| W14_1 | 111,075,916 | 90,398,208 (81.38%) | 97.55 | 93.68 | 50.24 |
| W14_2 | 93,593,136 | 76,315,647 (81.54%) | 97.70 | 94.00 | 50.72 |
| W14_3 | 84,971,878 | 68,209,772 (80.27%) | 97.63 | 93.83 | 50.86 |
| W22_1 | 96,542,790 | 77,999,491 (80.79%) | 97.20 | 92.88 | 53.12 |
| W22_2 | 91,219,510 | 74,510,394 (81.68%) | 97.48 | 93.57 | 53.00 |
| W22_3 | 108,973,446 | 88,507,667 (81.22%) | 97.60 | 93.77 | 52.03 |
| W30_1 | 103,147,962 | 73,578,615 (71.33%) | 97.20 | 92.88 | 53.87 |
| W30_2 | 85,885,112 | 66,663,484 (77.62%) | 96.48 | 91.06 | 51.51 |
| W30_3 | 92,626,188 | 73,938,322 (79.82%) | 96.90 | 92.05 | 51.54 |

The W6, W14, W22, and W30 indicate the breast muscle samples from 6, 14, 22, and 30 weeks, respectively.
