# Supplementary material for: Analyses of MicroRNA and mRNA Expression Profiles Reveal the Crucial Interaction Networks and Pathways for Regulation of Chicken Breast Muscle Development
Source: Front Genet. 2019 Mar 18;10:197. doi: 10.3389/fgene.2019.00197 (PMC6431651; doi:10.3389/fgene.2019.00197)
Supplement: Supplementary file 5 [file Table_5.docx]

**Table S5. Statistics for the small RNA library sequences of Gushi chicken breast muscle.**

| **Library name** | **Clean reads** | **Mapped sRNA** | **Q20(%)** | **Q30(%)** | **GC content (%)** |
| --- | --- | --- | --- | --- | --- |
| W6 | 13,972,867 | 12,528,764 | 97.61 | 95.84 | 44.37 |
| W14 | 12,242,777 | 10,564,852 | 96.91 | 94.43 | 44.96 |
| W22 | 12,725,882 | 11,558,618 | 97.47 | 95.47 | 43.96 |
| W30 | 12,457,614 | 10,793,441 | 96.62 | 93.61 | 44.76 |

The W6, W14, W22, and W30 indicate the breast muscle samples from 6, 14, 22, and 30 weeks, respectively.
